# 

**Published:** 1891-12-26

**Authors:** 


					INVALIDS
*>r INFANTS
'homas s H'ospitA.
lRW/CH HOSPI TAL
iLAScatv Western iNFmuARY
1 WITH EXTRA NURSING SUPPLEMENT.
Saturday, December 26th, 1891

				

